# Factors Affecting Sleep Quality of College Students during the Coronavirus Disease 2019 Pandemic: A Cross-Sectional Study

**DOI:** 10.3390/medicina59020416

**Published:** 2023-02-20

**Authors:** Mihyoung Kwon, Jihyun Oh

**Affiliations:** 1 Department of Nursing, College of Life and Health, Pai Chai University, Daejeon 35345, Republic of Korea; 2 Department of Nursing, College of Nursing and Health, Kongju National University, Kongju 32588, Republic of Korea

**Keywords:** sleep quality, fear of coronavirus disease 2019, uncertainty intolerance

## Abstract

*Background and Objectives:* The purpose of this study was to identify the correlation between college students’ fear of coronavirus disease 2019 (COVID-19), intolerance of uncertainty, and sleep quality during the COVID-19 pandemic, and to identify factors affecting sleep quality. *Materials and Methods:* Data were collected through an online survey of 310 college students from three universities located in three regions in Korea. *Results:* The average sleep quality score of college students was 4.76 ± 2.86 points, the average fear of COVID-19 was 14.01 ± 5.05 points, and the average intolerance of uncertainty was 31.50 ± 7.92 points. Fear of COVID-19 and intolerance of uncertainty were positively correlated (*r* = 0.302, *p* < 0.001). Sleep quality was positively correlated with fear of COVID-19 (*r* = 0.246, *p* < 0.001). Sleep quality was positively correlated with intolerance of uncertainty (*r* = 0.212, *p* < 0.001). Health status was the most powerful factor that affected sleep quality (β = 0.377, *p* < 0.001). The next most powerful factors that affected sleep quality were fear of COVID-19 (β = 0.164, *p* = 0.003) and intolerance of uncertainty (β = 0.122, *p* = 0.027), respectively. *Conclusions:* These results are expected to be used as basic data for the development of health intervention programs to protect and improve the psychological well-being of college students by improving their sleep quality.

## 1. Introduction

Over the past three years, people have been severely constrained by the coronavirus disease 2019 (COVID-19) pandemic in many areas of life, including society, the economy, and health, and therefore have had to deal with various threats [1,2,3,4]. In addition, in a situation where the end of the spread of infection was not predicted, the prolonged state of infection further exhausted people and negatively affected the mental health of society as a whole through fear of infection, anxiety, and depression [3,4,5]. The emergence of mutant viruses, the repeated re-proliferation of infectious diseases, and the progression of COVID-19, which has a high infection rate, amid uncertainty about when the infectious disease will end, have caused fear [6]. College students include both adolescents and adults. Therefore, in addition to adaptation as a college student, college students face various stressful situations as they take up roles they have not experienced before, position themselves as adults, establish their self-identity, and extend interpersonal relationships [7]. In other words, even in non-pandemic situations, college students already experience considerable mental health problems [8]. In addition, as the COVID-19 pandemic has caused serious disruptions in the implementation of programs across educational institutions, the most vulnerable group is students [9]. According to the government’s quarantine guidelines, wearing masks, implementing physical distancing, and restrictions to private gatherings have been leveraged to prevent the spread of COVID-19. Amid the COVID-19 crisis, school education has changed from a campus-centered education system to online classes, and as teaching methods and school guidelines continue to change depending on the situation, not only students’ uncertainty but also their fear has increased [8,9,10]. Fear is one of the most important factors that negatively affects mental health and well-being as a basic human emotion activated in response to perceived threats [11]. Fear is also a significant factor for students who have to perform their studies because it causes changes in certain functions in the cognitive domain, such as attention levels [12]. The COVID-19 pandemic has caused fear and educational confusion, including in the lives of college students, and these fears have been reported to have a negative impact on academic and professional aspirations [13,14]. Furthermore, considering that a Pew Research Center survey [15] found that Koreans perceive the spread of infectious diseases as a greater threat compared to other advanced countries, we think there is a great need to analyze the fears of Korean college students during the COVID-19 pandemic.

One of the effects of COVID-19 is uncertainty, which has been shown in previous studies to increase individual intolerance of uncertainty [14,16]. Intolerance of uncertainty is an important predictor of psychological well-being related to an individual’s mental health [16]. Intolerance of uncertainty is defined as the tendency to respond negatively to uncertain events in terms of cognitive, emotional, and behavioral aspects [17]. Buhr and Douglas [17] stated that intolerance of uncertainty has four structures that represent the idea that uncertainty causes stress, anger, and inability to act; that uncertain events are negative and should be avoided; and that being uncertain is unfair. Limited knowledge of the treatment of COVID-19, unpredictability of transmission, and confusion in daily and social life can all cause uncertainty [18]. Uncertainty is a major cognitive and psychological stress factor, and if not managed, it persists and affects emotional aspects, such as sleep disorders [18].

Several studies have shown that the incidence of decreased sleep quality and sleep disorders was higher during the COVID-19 pandemic [19,20,21,22]. College students who are prone to exposure to stressful situations are likely to perceive fear during the COVID-19 pandemic as stressful. Perceived stress hinders sleep quality [23]. Sleep is an essential component of human functioning [24]. Poor sleep quality is associated with increased susceptibility to viral infection, cognitive decline, poor work performance, and poor mental health [25]; as such, good sleep should be maintained. Identifying factors influencing the sleep quality of college students during the COVID-19 pandemic may help develop ways to intervene in sleep quality management.

The COVID-19 pandemic has created a wide range of problems in people’s psychological domains worldwide and is a threat to mental health. In this regard, although limited, research results dealing with COVID-19 and mental health problems have been published in various countries [3,4,5,11,26]. Among these, studies have been conducted on fear and uncertainty [10,16], fear and sleep quality [22], and uncertainty and sleep quality [18]; however, studies on fear, uncertainty, and sleep quality are difficult to find.

This study aimed to investigate the degree of fear, intolerance of uncertainty, sleep quality, and factors affecting sleep quality during the COVID-19 pandemic in college students. The results obtained in this study can be used as basic data for the development of health intervention programs to protect and improve the psychological well-being of college students by understanding the relationship between fear of COVID-19, intolerance of uncertainty, and sleep quality.

## 2. Materials and Methods

### 2.1. Study Design and Participants

A cross-sectional study was conducted to understand the correlation between fear of COVID-19, intolerance of uncertainty, and sleep quality and to identify factors affecting sleep quality among a convenience sample of college students in South Korea. The study participants were first-, second-, third-, and fourth-year college students in a bachelor’s degree program for diverse professional and academic objectives at three universities located in Ulsan City, Chungcheong-do, and Gyeongsang-do, South Korea. Individuals who had no difficulty in communication, had clear consciousness, were able to read and understand Korean for the questionnaire survey, and participated voluntarily were included. Those who were unable to read and understand Korean and those who were unable to communicate were excluded.

The number of participants required for this study was calculated using the G*Power 3.1.9.2 program [27], which is a sample size calculation program using Cohen’s formula. Using a significance level (a) of 0.05, power (1-β) of 95%, effect size (f) of 0.15, and 22 predictors for regression analysis, the minimum number of samples was calculated to be 230 participants. Considering the expected dropout rate and withdrawal from participation, 323 questionnaires were distributed. Ultimately, 310 appropriate questionnaires were analyzed, excluding 13 questionnaires due to incomplete responses.

### 2.2. Instruments

#### 2.2.1. Sleep Quality

Sleep quality was assessed using the Korean version of the Pittsburgh Sleep Quality Index (PSQI). Participants were instructed to complete the PSQI using a self-report questionnaire. The PSQI, developed by Buysse et al. [28], and the Korean version of the PSQI (PSQI-K), translated and modified by Sohn et al. [29], are self-rated questionnaires that assess subjective sleep quality and patterns over the past month. The PSQI-K questionnaire consists of 18 items with seven components. The seven components include subjective sleep quality, sleep latency, total sleep duration, habitual sleep efficiency, sleep disturbances, use of sleeping medication, and daytime dysfunction. Each item ranges from 0 to 3 points. In all cases, a score of 0 indicates no difficulty sleeping, and a score of 3 indicates serious difficulty sleeping. The total sum of the scores for the seven components ranges from 0 to 21 points, with a higher score indicating poorer sleep quality. A PSQI-K total score of <5 points indicates good sleepers, while a total score of ≥5 points indicates significantly bad sleepers, using the total PSQI-K score of 5 points as suggested by Buysse et al. [28]. In this study, Cronbach’s α was 0.86. 

#### 2.2.2. Intolerance of Uncertainty

Uncertainty intolerance was measured using the Korean version of the Intolerance of Uncertainty Scale short form (IUS-12) abbreviated by Carleton et al. [30] from the Intolerance of Uncertainty (IUS) form originally developed by Freeston et al. [31] and translated into Korean by Kim [32]. The IUS-12 consists of 12 items, including seven regarding perspective anxiety (e.g., I should always look ahead so as to avoid surprises) and five regarding inhibitory anxiety (e.g., I must get away from all uncertain situations). Each item is rated on a four-point Likert scale (1 = not at all, 4 = strongly agree). A higher score indicates a higher intolerance of uncertainty. The Korean versions of the IUS-12 have been validated, with a reported Cronbach α of 0.84 [32]. The overall reliability of the scale in this study was Cronbach’s α = 0.87, while the reliability of the subscales was Cronbach’s α = 0.78 for perspective anxiety and Cronbach’s α = 0.80 for inhibitory anxiety.

#### 2.2.3. Fear of COVID-19

The Fear of COVID-19 Scale (FCV-19S), developed by Ahorsu et al. [33], is a seven-item five-point scale that assesses fear of COVID-19 in the general population. This study used the Korean version translated by Seong et al. [34]. Each item is scored on a five-point scale where 1 stands for “never,” 2 for “rarely,” 3 for “moderately,” 4 for “often,” and 5 for “always.” The total score is the sum of the scores for each item and ranges from a minimum of seven points to a maximum of 35 points. A higher score indicates a higher fear of COVID-19. At the time of development, the internal consistency reliability of the scale was Cronbach’s α = 0.82; for the FCV-19S-K by Seong et al. [34], Cronbach’s α was 0.87, and for this study, Cronbach’s α was 0.92. 

### 2.3. Data Collection

The data collection period was from 5 July 2021 to 16 July 2021. Data were collected from 323 first-, second-, third-, and fourth-year college students at three universities via an online questionnaire survey. It took approximately 15 min for participants to complete the questionnaire. To ensure ethical data collection, the purpose, methods, efforts taken to protect of participants’ personal data, and time required for the survey were fully explained to the professors at the universities and the participants. 

### 2.4. Ethical Considerations

Before collecting data from university students, consent was obtained. Data collection was conducted after this study was approved by the Institutional Review Board (IRB) of Daejeon University (1040647-202102-HR-004-02). As this study involved students at universities not belonging to the researchers’ institution, permission was obtained from the heads of the universities in Ulsan City, Chungcheong-do, and Gyeongsang-do. After explaining the purpose and methods of this study to the professors concerned, students were informed about the necessity and methods of this study, and consent was obtained. Participants were selected through convenience sampling from those who agreed to participate. In addition, it was indicated that the collected questionnaires would be anonymized and not used for purposes other than this study. It was made clear that withdrawing from this study would be allowed at any stage, and there would be no disadvantage due to withdrawal. 

### 2.5. Data Analysis

The collected data were analyzed using SPSS WIN 27.0 (IBM Corp., Armonk, NY, USA). All continuous variables were confirmed for normal distribution before processing. Participants’ demographic characteristics were analyzed with descriptive statistics such as frequency and percentage. Fear of COVID-19, intolerance of uncertainty, and sleep quality were analyzed using mean and standard deviation. Differences in sleep quality, intolerance of uncertainty, and fear of COVID-19 according to the general characteristics were analyzed using the *t*-test and ANOVA, and a post-hoc test was performed using the Scheffé test. The correlation between fear of COVID-19, intolerance of uncertainty, and sleep quality was analyzed using Pearson’s correlation coefficients. Factors affecting sleep quality were identified using stepwise multiple regression analysis. Statistical significance was set at *p* < 0.05.

## 3. Results

### 3.1. General Characteristics of the Participants

In this study, the questionnaire was distributed to 323 participants, of whom 310, with the exception of 13 with incomplete information, were included in the analysis (response rate: 95.9%; Table 1). The mean age of the participants was 21.38 (±2.11) years. Most participants were female (86.5%) while 13.5% were male. The proportions of third—and first-year students were 30.6% and 30.0%, respectively. 

### 3.2. Sleep Quality, Intolerance of Uncertainty, and Fear of COVID-19 Levels

Table 2 shows the mean scores for each variable. 

### 3.3. Difference in PSQI, Intolerance of Uncertainty, and Fear of COVID-19 According to General Characteristics

Differences in sleep quality were analyzed according to the general characteristics of the participants. Regarding health status, unhealthy participants were the worst bad sleepers compared to healthy participants (*p* < 0.001). In addition, there was a difference in intolerance of uncertainty according to health status (F = 3.544, *p* = 0.030) (Table 3).

### 3.4. Correlation between Intolerance of Uncertainty, Fear of COVID-19, and Sleep Quality

Table 4 shows correlations between intolerance of uncertainty, fear of COVID-19, and sleep quality. The greater the fear of COVID-19, the worse the PSQI, and the more severe the intolerance of uncertainty, the worse the PSQI.

### 3.5. Factors Affecting Sleep Quality in the Participants

The results of verifying the assumptions of the regression analysis revealed that all assumptions of the regression equation were met. First, the results of testing for autocorrelation (independence) of errors revealed that the Durbin–Watson statistic was 1.905, indicating that there was no autocorrelation. Regarding the problem of multicollinearity, the tolerance was 0.297–0.961, which was above 0.1, and the variance inflation factor (VIF) was also 1.040–3.368, which did not exceed 10, indicating that there was no problem of multicollinearity. Finally, the linearity of the model, normality of the error term, and assumptions of homoscedasticity were met.

Therefore, to identify the major factors affecting sleep quality in university students based on the aforementioned results, intolerance of uncertainty, fear of COVID-19, and health status among general characteristics were analyzed using stepwise multiple regression analysis (Table 5). The results showed that the prediction model for sleep quality among university students was significant (F = 23.943, *p* = 0.000). The modified coefficient of determination (R^2^), which indicates the explanatory power of this model, was 0.182, and its explanatory power was 18.2%. 

The most powerful factor affecting sleep quality was health status (β =0.377, *p* < 0.001). The next most powerful factors affecting sleep quality were fear of COVID-19 (β = 0.164, *p* = 0.003) and intolerance of uncertainty (β = 0.122, *p* = 0.027), respectively. Among them, university students’ perceived health status was found to have the greatest effect on sleep quality.

## 4. Discussion

This study was conducted to understand the correlation between fear of COVID-19, intolerance of uncertainty, and sleep quality experienced by college students during the COVID-19 pandemic and to identify factors affecting sleep quality. The average sleep quality of the subjects was 4.76 points, and 34.2% of the subjects had poor sleep quality or a PSQI of 5 points or more. These results were similar to those of previous studies that evaluated the sleep quality of college students during the COVID-19 pandemic [9,35,36,37]. Several previous studies [9,22,35,36] have confirmed poor sleep quality during the COVID-19 pandemic. In addition, persistent sleep problems can have serious consequences, and our results suggest that college students need appropriate and active intervention to improve sleep quality in more than one-third of subjects, given that they are at a high risk of sleep disorders. In this study, fear of COVID-19 and sleep quality were compared by sex. The female score for fear of COVID-19 was higher than that of males, and the female score for sleep quality was higher than that of males. However, there was no significant difference according to sex. A study conducted by Doğanülkü et al. [14] on college students also found no significant difference in the degree of fear according to sex. However, our results differed from those of several previous studies that showed that women had a higher fear of COVID-19 than men [10,22,35,38] or that sleep quality was worse for women [20,21,22,35]. This study contradicts studies showing that female students are more psychologically vulnerable than male students. In general, women’s sleep quality was reported to be affected by hormones related to physiological reactions due to stress or hormones related to menstrual cycles [39,40,41], and women were found to have higher fear and lower sleep quality during COVID-19 than men [22,35]. The COVID-19 pandemic has been ongoing since December 2019. As data collection for our study took place a year and a half after the first outbreak of COVID-19, this may explain differences from the results of studies conducted at the beginning of the outbreak. Therefore, it is necessary to conduct additional investigations to confirm the factors influencing this difference.

In this study, subjects’ health status was measured through students’ indications of their subjective health status as healthy, moderate, or unhealthy. In general, it was found that subjects who perceived their health status as unhealthy had very poor sleep quality compared to subjects who perceived their health status as healthy, and health status perceived by college students had the greatest effect on college students’ sleep quality. These results are in line with the results of a study by Jahrami et al. [21], which showed that patients infected with COVID-19 are most affected by sleep problems and have a higher prevalence of sleep problems. During the COVID-19 pandemic, sleep is an important factor because it has many advantages not only for mental health but also for physical health [42]. Lack of sleep can make individuals more susceptible to the virus by compromising psychological function and decision-making and causing increased thinking, mood swings, increased medical costs, decreased concentration, and compromised immune responses [42]. In this respect, we found that maintaining good physical health in situations of new infectious diseases, such as COVID-19, is an important factor in maintaining sleep quality.

The results of this study showed that participants’ fear of COVID-19 and intolerance of uncertainty had a positive correlation. These results are similar to those of previous studies [10,16,43]. Previous studies have found that fear of COVID-19 predicts depression, anxiety, and stress, and the higher the level of fear of COVID-19, the higher the level of depression, anxiety, and stress responses [16,43]. As such, fear of COVID-19 has been the cause of individual psychological problems and negatively affects subjective well-being; the higher the level of fear and anxiety, the greater the intolerance to uncertainty [43]. COVID-19, which has strong transmission power, has caused a global epidemic, and uncertainty about the future has grown at a time when the number of reported cases and deaths is increasing [43]. Uncertainty increases the level of fear, and fear increases uncertainty [16,43]. Intolerance of uncertainty is associated with psychological problems; individuals with high intolerance of uncertainty experience emotional distress [44] and decreased academic achievement [45]. We believe that these results can be applied not only in situations such as the COVID-19 pandemic but also in various situations that can cause fear. Providing emotional support and interacting with subjects can be effective in reducing fear [46], and it is necessary to establish a framework that makes it easier for individuals with high levels of intolerance to receive more accurate information whenever necessary [14]. Universities need to develop intervention measures, such as programs to prevent or reduce psychological problems by managing college students’ fears and intolerance of uncertainty.

It was found that sleep quality had a positive correlation with fear of COVID-19 and intolerance of uncertainty. According to a study by De Los Santos et al. [9], college students’ increased fear of COVID-19 is associated with poor sleep quality. A previous study [22] stated that fear can stimulate the brain, cause sleep disorders due to excitement, and reduce cognitive as well as physical function, and studies have found that fear of COVID-19 is an important predictor of sleep quality. Sleep is an important factor for a person to restore their daily function and is essential to their ability to cope with emotional stress in everyday life [47]. In this respect, managing individual fear is very important. Research on the relationship between intolerance of uncertainty and sleep quality seems to have been limited. Therefore, comparisons between previous studies and our research results are limited. A study by Wu et al. [18] found that intolerance of uncertainty and sleep were not directly related, but uncertainty about COVID-19 was significantly positively correlated with intolerance of uncertainty and eventually led to poor sleep by increasing perceived stress.

This study found that intolerance of fear and uncertainty about COVID-19 had a significant effect on the quality of college students’ sleep. These results support the findings of a study by Vandekerkhove and Wang [47] that fear affects sleep physiology by causing emotional instability. This is consistent with the results of a study conducted by Colvonen et al. [48], which showed that serious levels of fear affect individual psychological well-being and reduce sleep quality. As a factor affecting sleep quality, a large amount of research on intolerance of uncertainty has not been conducted so far. In a limited study by Wu et al. [18], intolerance of uncertainty was found to be mediated by perceived stress, although it was not a factor that directly affected sleep. This suggests that it is necessary to intervene to ensure that intolerance of uncertainty does not negatively affect sleep quality.

### Limitations

This study had several limitations. First, since the investigation was conducted approximately a year and a half after the COVID-19 outbreak, the results may differ from studies conducted at the beginning of the outbreak, and the influence of various factors may change depending on the investigation period. Second, as a cross-sectional study, progress or change cannot be measured, and there are limits to identifying causal relationships. Third, there are limits to generalizing the research results to all college students because the subjects were sampled conveniently at some universities in several regions of Korea. Fourth, COVID-19 infection and isolation were not controlled. Fifth, only the relationship between limited variables was investigated; variables such as academic success, which may affect the subjects as they are college students, were not considered. Therefore, it is possible to increase the generalizability of results by conducting follow-up studies on college students with different cultural characteristics of different cultures and countries. Sixth, sleep quality evaluation has limitations in measuring sleep quality because the overall content of sleep was subjectively assessed and self-reported, Therefore, in order to accurately evaluate the quality of sleep, it is necessary to consider objective sleep evaluation. Future studies should consider these limitations, and longitudinal studies should be conducted to evaluate changes in fear of COVID-19, intolerance of uncertainty, and sleep quality. In addition, it is necessary to conduct a follow-up study that further expands the scope of variables and subjects.

## 5. Conclusions

This study found that factors affecting the sleep quality of college students during the COVID-19 pandemic were health status, intolerance of uncertainty, and fear of COVID-19. College students are vulnerable to mental health problems. Therefore, health problems must be recognized and managed early. The results of this study confirm the importance of active intervention in managing intolerance of fear and uncertainty. Our results are also expected to be used as important data for developing programs to improve the mental health and sleep quality of college students and to provide an approach to alleviating students’ vulnerability to these health problems.

## Figures and Tables

**Table 1:** Descriptive Statistics (*N* = 310).

Variable	*N* (%)	Mean ± SD
Mean Age (years) (range)		21.38 ± 2.11 18–33
Sex		
Male	42 (13.5)	
Female	268 (86.5)	
Grade		
1	93 (30.0)	
2	38 (12.3)	
3	85 (30.6)	
4	84 (27.1)	
Religion		
Yes	108 (34.8)	
No	202 (65.2)	
Smoking		
Yes	10 (3.2)	
No	300 (96.8)	
Alcohol		
Yes	215 (69.4)	
No	95 (30.6)	
Health status		
Healthy	176 (56.8)	
Moderate	93 (30.0)	
Unhealthy	41 (13.2)	
Good sleepers (PSQI < 5)	204 (65.8)	
Poor sleepers (PSQI ≥ 5)	106 (34.2)	

Note. PSQI, Pittsburgh Sleep Quality Index; COVID-19, Coronavirus Disease 2019.

**Table 2:** Degree among PSQI, Intolerance of Uncertainty, and Fear of COVID-19 (*N* = 310).

	Mean ± SD
Total PSQI scores	4.76 ± 2.86
Subjective sleep quality	0.96 ± 0.59
Sleep latency	1.35 ± 1.05
Sleep duration	0.29 ± 0.68
Habitual sleep efficiency	0.29 ± 0.73
Sleep disturbance	0.89 ± 0.63
Daytime functioning	0.90 ± 0.92
Sleep medication	0.05 ± 0.30
Intolerance of Uncertainty Scale	31.50 ± 7.92
Prospective IU	20.37 ± 4.94
Inhibitory IU	11.12 ± 3.74
Fear of COVID-19 Scale	14.01 ± 5.05

Notes. SD, standard deviation; PSQI, Pittsburgh Sleep Quality Index; IU, Intolerance of Uncertainty Scale.

**Table 3:** Difference in PSQI, Intolerance of Uncertainty, and Fear of COVID-19 according to General Characteristics (*N* = 310).

Variable	Total PSQI Score		Intolerance of Uncertainty		Fear of COVID-19 Scale	
	Mean ± SD	t or F *(p)* Scheffé	Mean ± SD	t or F *(p)* Scheffé	Mean ± SD	t or F *(p)* Scheffé
Mean Age (years) (range)						
Sex						
Male	4.28 ± 2.54	−1.208 (0.288)	65.30 ± 14.59	−0.690 (0.491)	13.14 ± 5.44	−1.209 (0.228)
Female	4.86 ± 2.96		67.27 ± 17.53		14.15 ± 4.99	
Grade						
1	4.94 ± 2.64	2.014 (0.112)	64.56 ± 15.27	0.932 (0.425)	14.69 ± 5.16	1.693 (0.169)
2	5.07± 3.19		67.55 ± 16.00		14.84 ± 4.74	
3	5.31 ± 3.27		68.48 ± 20.30		13.26 ± 5.16	
4	4.39 ± 2.56		67.79 ± 15.69		13.75 ± 4.88	
Religion						
Yes	4.65 ± 2.75	−0.587 (0.558)	67.03 ± 17.06	0.020 (0.984)	14.09 ± 5.14	0.186 (0.852)
No	4.86 ± 2.99		66.99 ± 17.24		13.98 ± 5.02	
Smoking						
Yes	5.10 ± 4.17	0.341 (0.733)	64.40 ± 16.69	−0.488 (0.626)	15.20 ± 3.88	0.750 (0.454)
No	4.78± 2.87		67.09 ± 17.19		13.98 ± 5.09	
Alcohol						
Yes	4.91 ± 2.71	−1.146 (0.253)	67.11 ± 16.24	−0.164 (0.870)	14.13 ± 5.19	−0.580 (0.562)
No	4.50 ± 3.32		66.76 ± 19.15		13.76 ± 4.74	
Health status						
Healthy ^a^	4.07 ± 2.47	24.074 (<0.001)	65.31 ± 17.98	3.544 (0.030)	13.45 ± 4.86	2.596 (0.076)
Moderate ^b^	5.02 ± 2.67	c > b > a	67.53 ± 14.56		14.68 ± 5.12	
Unhealthy ^c^	7.31 ± 3.67		73.09 ± 17.87		14.92 ± 5.53	

Notes. a = self-perceived health status: good, b = self-perceived health status: moderate, c = self-perceived health status: bad.

**Table 4:** Correlations among Fear of COVID-19, Intolerance of Uncertainty, and PSQI (*N* = 310).

	1. Fear of COVID-19 Scale	2. Intolerance of Uncertainty Scale	3. Prospective IU	4. Inhibitory IU	5. Global PSQI Scores	6. Subjective Sleep Quality	7. Sleep Latency	8. Sleep Duration	9. Habitual Sleep Efficiency	10. Sleep Disturbance	11. DayTime Functioning	12. Sleep Medication
1	-											
2	0.302 **	-										
3	0.231 **	0.935 **	-									
4	0.333 **	0.883 **	0.658 **	-								
5	0.246 **	0.212 **	0.182 **	0.210 **	-							
6	0.188 **	0.166 **	0.122 *	0.190 **	0.698 **	-						
7	0.111	0.053	0.047	0.051	0.695 **	0.474 **	-					
8	0.110	0.091	0.092	0.070	0.532 **	0.266 **	0.143 *	-				
9	0.101	0.040	0.008	0.075	0.524 **	0.213 **	0.198 **	0.366 **	-			
10	0.152 **	0.135 *	0.118 *	0.131 *	0.603 **	0.430 **	0.380 **	0.129 *	0.108	-		
11	0.226	0.287 *	0.279 **	0.240 **	0.597 **	0.331 **	0.189 **	0.190 **	0.135 *	0.259 **	-	
12	0.150	0.041	−0.008	0.097	0.019	−0.008	0.017	0.026	0.008	0.093	−0.019	-

* *p* < 0.05, ** *p* < 0.01.

**Table 5:** Results of the stepwise multiple regression analysis for Total PSQI (*N* = 310).

Variables	Total PSQI			
	β	95% Confidence Interval	*t*	*p*
Constant	0.312 (0.597)	−0.862, 1.486	0.522	0.602
Health status	0.377	0.939, 1.758	6.484	<0.001
Fear of COVID-19 Scale	0.164	0.032, 0.154	2.994	0.003
Inhibitory IU	0.122	0.011, 0.175	2.225	0.027
R^2^	0.190			
Adjusted R^2^	0.182			
df	3			
F	23.943			<0.001

Note. PSQI = Pittsburgh Sleep Quality Index.

## Data Availability

The data presented in this study are available on request from the corresponding author. The data are not publicly available owing to privacy.
